# EEG-based driving intuition and collision anticipation using joint temporal-frequency multi-layer dynamic brain network

**DOI:** 10.3389/fnins.2024.1421010

**Published:** 2024-06-26

**Authors:** Jialong Liang, Zhe Wang, Jinghang Han, Lihua Zhang

**Affiliations:** ^1^Academy for Engineering and Technology, Fudan University, Shanghai, China; ^2^Engineering Research Center of AI and Robotics, Fudan University, Shanghai, China; ^3^School of Data Science, Fudan University, Shanghai, China

**Keywords:** driving intuition, multi-layer networks, functional connectivity, PLI, EEG

## Abstract

Intuition plays a crucial role in human driving decision-making, and this rapid and unconscious cognitive process is essential for improving traffic safety. We used the first proposed multi-layer network analysis method, “Joint Temporal-Frequency Multi-layer Dynamic Brain Network” (JTF-MDBN), to study the EEG data from the initial and advanced phases of driving intuition training in the theta, alpha, and beta bands. Additionally, we conducted a comparative study between these two phases using multi-layer metrics as well as local and global metrics of single layers. The results show that brain region activity is more stable in the advanced phase of intuition training compared to the initial phase. Particularly in the alart state task, the JTF-MDBN demonstrated stronger connection strength. Multi-layer network analysis indicates that modularity is significantly higher for the non-alert state task than the alert state task in the alpha and beta bands. In the W4 time window (1 second before a collision), we identified significant features that can differentiate situations where a car collision is imminent from those where no collision occurs. Single-layer network analysis also revealed statistical differences in node strength and local efficiency for some EEG channels in the alpha and beta bands during the W4 and W5 time windows. Using these biomarkers to predict vehicle collision risk, the classification accuracy of a linear kernel SVM reached up to 87.5%, demonstrating the feasibility of predicting driving collisions through brain network biomarkers. These findings are important for the study of human intuition and the development of brain-computer interface-based intelligent driving hazard perception assistance systems.

## 1 Introduction

Intuition, as an essential cognitive process in human decision-making and problem-solving, has been extensively described and researched across multiple disciplines, including philosophy (Bealer, 1998), psychology (DePaul and Ramsey, 1998), management (Akinci and Sadler-Smith, 2012), and cognitive science (Nichols, 2004). Intuition involves rapid judgment and processing of information, often occurring at a subconscious level. In psychology, intuitive decision-making is viewed as a swift cognitive process playing a key role in handling complex situations. Slovic and Västfjäll (2010) proposed that in hazardous situations, decisions are made through an automatic processing system reliant on emotions and experience, which is mostly irrational and faster than controlled processing systems. According to Daniel Kahneman's dual-system theory (Daniel, 2017), intuitive decision-making is usually associated with the brain's System 1 thinking, characterized by being fast, automatic, and requiring little cognitive resources. In contrast, System 2 thinking is slower, more logical, and conscious. Moreover, the effectiveness of intuition varies in different application scenarios: experienced pilots can make quick and accurate decisions in adverse weather, seasoned police officers can rapidly identify suspects, and skilled table tennis players can accurately anticipate the ball's landing point and direction in a short time (Cokely and Feltz, 2014). It is this rapid and unconscious decision-making process that plays a vital role in enhancing driving safety.

Driving intuition is a typical manifestation of human brain intuition in real-world scenarios and is an important focus for studying the emergence and development of intuition in complex environments (Risen, 2017). Driving, a daily activity fraught with risks of injury, death, and associated costs, demands high levels of cognitive and sensory engagement from drivers (Abay and Mannering, 2016). Although many manage to maintain safety, the complexity and variability of the driving environment continually pose potential risks. In such contexts, human intuition is pivotal, particularly in collision anticipation. It emerges as a complex cognitive process, where drivers often take preventative actions, like quick braking or steering, before fully realizing the risk (Duma et al., 2017). This preconscious warning in driving scenarios, crucial for emergency responses, might be key in reducing traffic accidents. Recent studies, including those by Liang and Lin (2018), have showed that classical drivers' risk and safe classification could very well be done by using physiological and behavioral measures. Concurrently, Zhang and Yan (2023) has leveraged these EEG indicators to develop a neural network model that estimates collision probabilities at unsignalized intersections.

Existing research has confirmed that the human brain can perceive potential risks before the arrival of danger, a phenomenon that underscores the importance of intuition in driving safety. For example, the study by Kveraga highlights the brain's ability to use past experiences to interpret sensory information and predict the future, particularly in visual recognition (Kveraga et al., 2007). Furthermore, research by Shankaran demonstrates that the brain's response to fear is so rapid that it occurs before conscious recognition, a finding confirmed through the study of the amygdala using ultra-high field magnetic resonance imaging techniques (Ravi Shankaran, 2013). Steingroever et al. (2018)'s study highlights intuitive decision-making mechanisms. Comparing healthy individuals and frontal lobe-damaged patients in the Iowa Gambling Task, they find that healthy participants develop an ability to anticipate and avoid card decks previously associated with net losses (Steingroever et al., 2018). Moreover, the Contingent Negative Variation (CNV), an endogenous component associated with cognition discovered by Walter in 1964, has been linked to event anticipation and motor preparation. Analysis of its waveform composition and brain electrical signals related to prediction has provided new insights into this field (Cohen, 1969; Berchicci et al., 2020; Kóbor et al., 2021; Fiorini et al., 2023). These studies reveal that human intuitive responses in decision-making are driven by complex brain mechanisms, especially in high-risk and rapid-response scenarios such as driving. This involves the coordinated work of brain, including sensory information processing (Frolov et al., 2019), memory retrieval (Rugg and Vilberg, 2013), risk assessment (Brandtstädter et al., 2004; Li et al., 2022), and predicting future events (Ramnani and Miall, 2004; Mullally and Maguire, 2014), enabling drivers to react quickly in emergencies.

### 1.1 EEG-based functional brain network

With its high temporal resolution and the ability to precisely track neural activity, electroencephalography (EEG) has become a powerful tool for exploring the brain's working state. Especially in improving assisted driving systems, EEG has sparked research interest, such as in driving fatigue detection (Mu et al., 2017; Ma et al., 2019), distraction detection (Li et al., 2021; Zuo et al., 2022), and more, providing important foundations for developing driving safety systems. Additionally, with its temporal resolution far surpassing that of functional Magnetic Resonance Imaging (fMRI), EEG plays a key role in neuroscience research on the brain's decision-making process in fast, unconscious states (Mullinger and Bowtell, 2011). Recent neuroscience studies indicate that the brain's executive capabilities stem not just from the independent roles of distinct regions, but also from their interconnectivity and communication (Stallen and Sanfey, 2015; Lu et al., 2019). Likewise, intuition, a key cognitive function, depends significantly on the interconnectedness and cooperation of brain regions (Kuo et al., 2009; Erdeniz and Done, 2019).

Functional brain networks constructed using EEG serve as an invaluable methodology for the investigation of interdependencies among various cerebral regions (van Straaten and Stam, 2013). These networks facilitate the precise identification and analysis of interconnected and active cerebral regions during intuitive driving, thereby yielding pivotal insights into the neural mechanisms underlying this phenomenon. Currently, this approach has seen extensive application in research pertinent to driving. Notably, Wang observed alterations in the optimal topological structure of the Phase Lag Index (PLI) functional network amidst driving fatigue, with a particular emphasis on the connectivity alterations from frontal to parietal or occipital regions (Wang et al., 2021b). In parallel, Perera et al. (2022) investigated EEG-based driver distraction classification, employing diverse brain connectivity estimation methods. In addition to driving, the combined application of functional brain networks and graph theory analysis has been extended to other scientific fields, including the diagnosis and treatment of neurological disorders (Jiao et al., 2023; Zeng et al., 2024), motor imagery (Gu et al., 2020), and emotion recognition (Guo et al., 2024), thereby underscoring their extensive scientific merit and potential for varied applications.

Current research on the neural mechanisms underlying intuition, particularly in driving, remains limited. The majority of extant research primarily focuses on discerning intuitive predictive behaviors by comparing statistical significances in the time-frequency domains of various EEG signals, which is limited to establishing probabilistic disparities at the level of EEG signals and lacks a robust physiological interpretability (Duma et al., 2017; Jia et al., 2023). Moreover, several studies are limited to the correlation between isolated cerebral region and intuition, thereby neglecting the crucial interplay among different cerebral regions (Kuo et al., 2009; Erdeniz and Done, 2019). Notably, intuition is a dynamic cognitive process involving rapid collaboration and reorganization among cerebral regions, and it can be enhanced through specific training (Fellnhofer et al., 2023). However, most related research focuses on brain activity at fixed time period lengths, neglecting the dynamics of cerebral region activities over time and the individual variability and learnability of intuitive capabilities, thereby failing to explore the temporal evolution of brain connectivity. To address these limitations, a novel approach has emerged: the multi-layer dynamic networks (Han et al., 2020; Chang et al., 2022). This analytical method measures the synchrony of EEG signals across different time windows or bands, integrating the advantages of single-layer networks while emphasizing dynamic characteristics in the temporal dimension.

To address extant gaps in the field, this study represents the first systematic integration of driving intuition, collision anticipation, and dynamic networks. Employing a combination of PLI and the innovative JTF-MDBN approach, we investigated the brain region connectivity changes corresponding to perception, prediction, and response in instantaneous vehicle collision scenarios. By analyzing the dynamic characteristics of brain networks during the initial (ITIP) and advanced phase (ITAP) of intuition training, this research reveals the patterns of brain network activity in situations of emergency evasion or impending collision, identifying significant changes in the brain network during emergency responses. These findings offer new perspectives on understanding the dynamics of brain networks during driving and provide a scientific basis for the development of EEG-based collision anticipation systems and Advanced Driver-Assistance Systems (ADAS). The key contributions of this paper are as follows:

1. Provided a detailed examination of the brain network characteristics during the driving intuition process by analyzing dynamic networks, particularly highlighting the dynamic changes in brain networks at moments of emergency evasion and potential collision.
2. Conducted a comparative analysis of the brain network differences between the initial and advanced phases of intuition training, demonstrating that specific training or stimuli can enhance the effectiveness of intuition.
3. Integrated and evaluated a suite of biomarkers, encompassing multi-layer and single-layer network features(both local and global), substantiating the efficacy of driving intuition biomarkers in collision identification through classification testing.

## 2 Materials and methods

### 2.1 Public datasets

We utilized a publicly available EEG dataset named the Simulated Car Crash Anticipation EEG Dataset (SCCA EEG Datasets) (Duma et al., 2017), which focuses on danger perception in intuitive driving. This dataset was collected using a simplified driving simulator as a behavioral task, capturing EEG signals of participants during the process of intuitive driving. The dataset includes EEG recordings from 40 participants using a 32-channel EEG device based on the 10–20 international system (electrode channel positions are shown in Supplementary Figure 1). In the original study, each participant was involved in two task states: “Non-Alert State” (NAS) and “Alert State” (AS).

During the NAS task, participants were required to watch a segment of driving simulation to familiarize themselves with the environment and establish a temporal expectation, with explicit notification that no collisions would occur during this phase. The NAS task consisted of 14 trials, each lasting 7 to 10 seconds. In the AS task, the task involved two possible outcomes: “CrashEnd” (AS-CE) and “NoCrash” (AS-NC). Participants were informed about the randomness of the trial endings and were asked to make an effort to predict whether a car crash would occur. The AS task involved 20 trials each time, with durations randomly varying between 25 to 40 seconds. Collisions occurred randomly in 50 % of the trials.

### 2.2 EEG preprocessing

In this study, we employed standardized preprocessing steps to reduce noise interference and used signals from the pre-event period to correct for EEG responses during the studied events, thereby controlling for pre-existing differences in brain activity unrelated to the experimental conditions. The preprocessing of EEG data was accomplished through custom programming in MATLAB. The first step in preprocessing involved downsampling the data to a sampling rate of 256 Hz. This was followed by a series of filtering operations targeted at three distinct frequency ranges: theta band (4–8 Hz), alpha band (8–13 Hz), and beta band (13–30 Hz) using finite impulse response (FIR) filters to minimize phase distortions. After filtering, the data underwent further cleaning and formatting, which included the removal of outer channels in the EEG recordings. Additionally, the data was segmented into periods ending with specific events, each with a duration of three seconds, to capture brain activity preceding the occurrence of these events.

Independent Component Analysis (ICA) was applied to identify and remove artifacts using the EEGLAB (Delorme and Makeig, 2004), plugins FASTER (Nolan et al., 2010), and ADJUST (Mognon et al., 2011), where FASTER was utilized for initial automatic artifact detection and ADJUST for fine-tuning artifact removal based on statistical thresholds. Post-ICA, channels containing artifacts underwent interpolation to ensure data integrity and consistency, followed by average reference processing to reduce common noise and enhance data quality. Finally, to investigate the two phases of intuition training, ITIP and ITAP, the study defined the first five experimental trials as ITIP and the last five trials as ITAP.

### 2.3 Construction of EEG network

#### 2.3.1 Network construction and sliding time window

PLI is a phase-based method for analyzing functional connectivity, utilized to assess phase synchronization between signals from two channels (Stam et al., 2007). This PLI metric is particularly apt for exploring functional connectivity in multi-channel EEG data. It mitigates the volume conduction effects often encountered in EEG signal acquisition, thereby more accurately reflecting true connectivity between brain regions.


(1) $$\text{PLI}=\langle \text{sign}\left(\Delta \phi_{\text{rel}}(\text{t})\right)\rangle \rangle =|\frac{1}{N}\Sigma_{\text{n}=1}^{\text{N}}\text{sign}\left(\Delta \phi_{\text{rel}}({\text{t}}_{\text{n}})\right)$$

Where *n* represents the time points, and Δϕ_*t*_ denotes the relative phase difference between two signals at time *t*. The instantaneous phase is calculated using the Hilbert transform. The sign of this phase difference, whether positive, negative, or zero, is determined using the signum function, sign. The PLI is the average of the signs of the phase differences across all time points. Consequently, the PLI value ranges from 0 to 1. A value close to 0 indicates a lack of consistent phase lead or lag relationship between the two signals.

**Figure 1:**
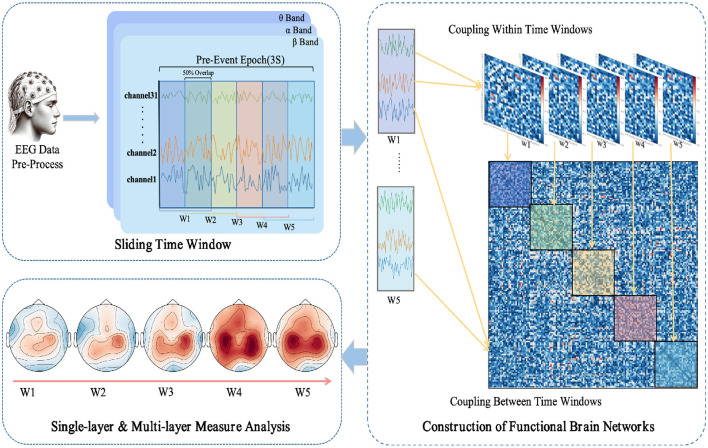
Workflow for EEG data processing and multi-layer dynamic network analysis in anticipation of driving intuition.

The use of a sliding window method aims to parse the temporal variability in EEG signals, thereby revealing the dynamic integration and reconfiguration processes of the brain's functional networks (O'Neill et al., 2018). As depicted in Figure 1, each task epoch, lasting three seconds, is divided into time windows of one-second width with a step size of 0.5 seconds, resulting in a total of five time windows (W1 to W5). This choice is based on previous research (Chang et al., 2022) and empirical evidence, indicating that these parameters strike a balance between temporal resolution and computational feasibility. For each time window, the PLI values are computed between pairs of EEG channels, yielding a symmetric functional connectivity matrix.

#### 2.3.2 Construction of multi-layer networks

Multi-layer networks, integrating multiple single-layer networks, can reveal deeper insights that are not discernible in single-layer networks (Hipp et al., 2012; Boccaletti et al., 2014). In such a structure, each layer shares the same set of nodes, representing different regions of the brain. This configuration encompasses not only the connectivity information among nodes within each network but also includes the connections across different layers. The focus of our analysis will be on the characteristics of the weighted networks both within and between each layer of this multi-layer network structure.

In constructing multi-layer networks, we use PLI to quantify inter- and intra-layer connectivity. For intra-layer connectivity, PLI calculates the likelihood of synchronization between pairs of EEG channels within the time window, thus encompassing the functional connectivity within each different network layer. In contrast, for inter-layer connectivity, PLI evaluates the synchronization between different time windows, thus providing insight into the dynamic interactions between the layers of the network.

To precisely describe the multi-layer network structure in mathematical terms, the concept of a supra-adjacency matrix is introduced (Boccaletti et al., 2014). Specifically, the supra-adjacency matrix can be defined by the following formula:


(2) $$A_{supra}=\text{diag}(A_{l})+(H⊗B)$$

Where diag(**A**_*l*_) refers to a block diagonal matrix. This block diagonal matrix is composed of the adjacency matrices **A**_*l*_ for each layer *l* within the multi-layer network, where *l* ranges from 1 to *L*. On the other hand, (**H** ⊗ **B**) represents the connections between layers, where **H** is a matrix describing the strength and pattern of connections between different layers, **B** represents a matrix that defines the pattern of inter-layer connections. The symbol ⊗ indicates the Kronecker product. In this study, **B** is set as **E**, where **E** is a matrix composed entirely of ones. Therefore, the matrix constructed in this manner, incorporating both intra-layer and inter-layer connections, can be represented as follows:


(3) $$\begin{array}{l} {\text{A}}_{\text{supra}}=\left[\begin{array}{cccc} {\text{A}}_{1} & {\text{H}}_{12} & \ldots & {\text{H}}_{1\text{L}}\\ {\text{H}}_{21} & {\text{A}}_{2} & \ldots & {\text{H}}_{2\text{L}}\\ \vdots & \vdots & \ddots & \vdots \\ {\text{H}}_{\text{L}1} & {\text{H}}_{\text{L}2} & \ldots & {\text{A}}_{\text{L}} \end{array}\right] \end{array}$$

#### 2.3.3 Multi-layer network measures

In the metric analysis of multi-layer networks, this paper primarily employs three characteristic parameters. Firstly, to reveal the coordination consistency between different layers, multi-layer modularity is a concept quantified by the Q-value. The Q-value varies from 0 to 1, representing the degree of network separation from low to high. It signifies the level of separation between different layers (Mucha et al., 2010; Pedersen et al., 2018). The definition of Q-value in a multi-layer context is outlined as follows:


(4) $$\begin{array}{l} Q(\gamma ,\omega )=\\ \frac{1}{2\mu }\sum_{ijlm}[(A_{l}-\gamma_{l}\frac{k_{is}k_{jl}}{2m_{l}})\delta (A_{il},A_{jl})+\delta (i,j)\cdot \omega_{jml}]\delta (A_{il},A_{jm}) \end{array}$$

Where γ signifies the cumulative link strength within the multi-layer network. *k* represents the strength of a node *i* at the layer *l*, and *m* denotes the total degree sum of all nodes at the same layer *l*. γ_*l*_ denotes the resolution parameters specific to the topology of the layer *l*, and ω_*jml*_ symbolizes the inter-temporal connectivity parameter for node *j* across layers *l* and *m*. δ(**A**_*il*_, **A**_*jm*_) are 1 for nodes in the same module and 0 otherwise.

To further investigate the brain's integrative and coordinative functions under various conditions, the concept of Multi-layer Participation Coefficient (MPC) is introduced (Boccaletti et al., 2014). It measures the homogeneity of the number of neighbors a node has within a multi-layer network. It is calculated as follows:


(5) $$\begin{array}{l} \text{MPC}=\frac{\overset{N}{\underset{i=1}{\sum }}{\text{MPC}}_{i}}{N}=\frac{\text{L}}{\text{L}-1}\left[1-\overset{\text{L}}{\underset{l=1}{\sum }}{\left(\frac{{\text{k}}_{i}^{\left[l\right]}}{{\text{o}}_{i}}\right)}^{2}\right] \end{array}$$

where *L* is the number of layers. *MPC*_*i*_ represents the *MPC*_*i*_ value of node *i* within a multi-layer network. *MPC*_*i*_=1 when the degree is the same in all layers and *MPC*_*i*_=0 when a node has non-zero degree in only one layer. $k_{i}^{\left[l\right]}$ is the degree in the layer *l*. *o*_*i*_ is the overlapping degree of the node *i*. The mean *MPC* for the entire multiplex network is calculated by averaging the individual *MPC*_*i*_ values across all nodes.

In addition to the previously mentioned metrics, the multi-layer network can also be evaluated using Layer-Layer Correlation (LLC) Coefficients (Boccaletti et al., 2014). This involves computing the Pearson correlation coefficient between every pair of network layers to assess the degree of correlation among the layers, particularly between layers corresponding to different time windows. The formula for LLC is as follows:


(6) $$R(M_{i},M_{j})=\frac{\sum \left(M_{i}-\bar{M_{i}}\right)(M_{j}-\bar{M_{j}})}{\sqrt{\sum {\left(M_{i}-\bar{M_{i}}\right)}^{2}\sum {\left(M_{j}-\bar{M_{j}}\right)}^{2}}}$$

Where **M**_*i*_ and **M**_*j*_ represent the matrix blocks from different time windows in the super-adjacency matrix. $\bar{{\text{M}}_{i}}$ and $\bar{{\text{M}}_{j}}$ are the mean values of the matrix blocks **M**_*i*_ and **M**_*j*_, respectively.

#### 2.3.4 Single-layer network measures

In the analysis of single-layer networks, we employed two types of classic graph theory metrics. These metrics are crucial for revealing the complex structure and functional characteristics of brain networks (Rubinov and Sporns, 2010). Local metrics delve into the properties of individual nodes or small groups of nodes within the network. *Node strength (NS)* is defined as the sum of the weights of all edges connected to that node. *Path length (PL)* is the average distance from one node to all other nodes, reflecting the efficiency of information transfer in the network. *Local efficiency (E-loc)* reflects the compactness of the “group” of adjacent nodes in the network, and is defined as the harmonic mean of the shortest path between all nodes in the sub-network. *Betweenness centrality (BC)* is the proportion of all the shortest paths in the network that pass through a given node, with nodes having higher BC values participating in a large number of shortest paths. *Eigenvector centrality (EC)* takes into account not only the number of connections of a node but also the importance of its neighbors, with higher values indicating that the node is a key player in information transfer and integration. Global indices provide a macroscopic understanding of the performance and characteristics of the network as a whole. The *Clustering Coefficient (CC)* of a node is defined as the ratio of the number of existing connections among the node's neighbors to the maximum possible number of edges between them. The *Assortativity (Ass)* coefficient refers to the correlation coefficient between the strengths of the nodes at both ends of an edge, used to measure the correlation between connected pairs of nodes. The calculation methods for single-layer network metrics are shown in Supplementary material.

#### 2.3.5 Statistical methods

Before the main analysis, we verified that our data met the prerequisites for normal distribution and variance homogeneity required by ANOVA. We conducted the Shapiro-Wilk test for normality and Levene's test for equality of variances. Upon confirming that the data met these assumptions, we proceeded with one-way ANOVA to examine differences in multi-layer network metrics, connectivity strengths, and individual single-layer network characteristics across the three task conditions (NAS, AS-CE, and AS-NC). Given the multiple metrics and time windows analyzed, we applied False Discovery Rate (FDR) correction to ensure that the reported effects are robust statistically. Additionally, a single “*” indicates a corrected *p-*value less than 0.05, “**” denote a *p*-value less than 0.01, and “***” represent a *p*-value less than 0.001.

## 3 Results

### 3.1 Connectivity of multi-layer networks

**Figure 2:**
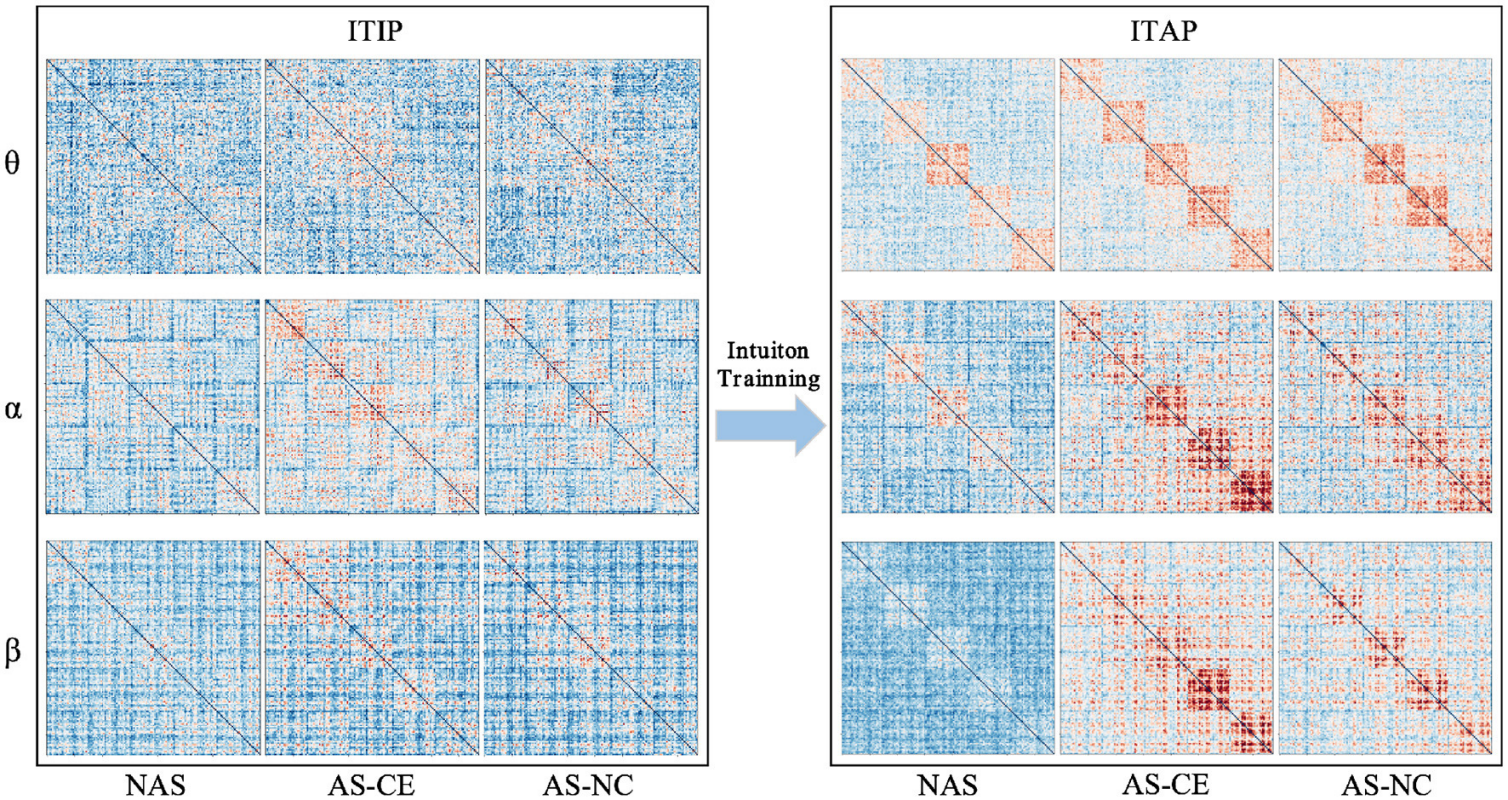
PLI brain network connectivity during initial and advanced phases of intuition training (ITIP vs. ITAP).

Figure 2 illustrates the changes in functional connectivity during intuitive driving, specifically presenting the multi-layer network super-adjacency matrices across theta, alpha, and beta bands in three task states (NAS, AS-CE, and AS-NC) for both ITIP and ITAP. These matrices are derived from the average of all trial data, with diagonal blocks representing coupling within time windows and off-diagonal blocks revealing coupling between time windows.

The analysis indicates that the PLI connectivity strength within the central diagonal matrix blocks is significantly higher than in the off-diagonal blocks. Notably, during the ITIP, the connectivity patterns observed in the ITAP were absent, suggesting that the brain network during ITIP had not yet formed a stable pattern, rendering it unsuitable for intuition studies. In the ITAP, regardless of theta, alpha, or beta bands, intra-layer connectivity was observed to be markedly stronger than inter-layer connectivity. This was particularly evident in the NAS task, where connectivity levels were significantly lower than in the AS task, including both AS-CE and AS-NC conditions. In AS, enhanced intra- and inter-layer connectivity was observed in time windows W3, W4, and W5. Given these findings, subsequent analyses will primarily focus on the brain networks in the ITAP.

**Figure 3:**
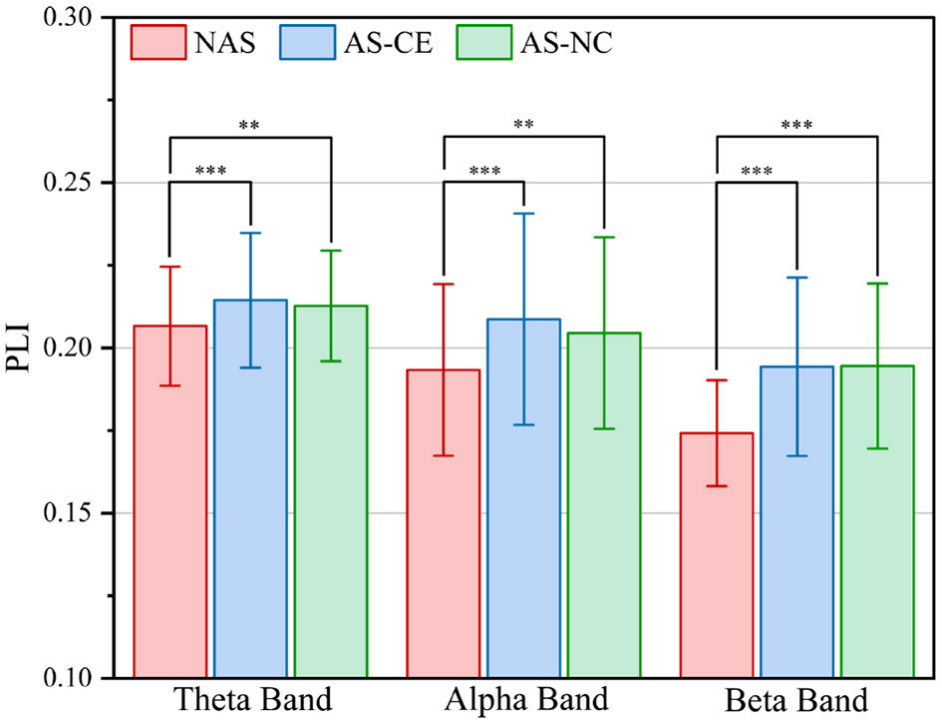
Grand averaged PLI values across task conditions in theta, alpha, and beta bands. **p-value < 0.01, ***p-value < 0.001.

To comprehensively assess the variations in network connection weights during the ITAP under different conditions, we calculated the overall mean of the supra-adjacency matrices for three different experimental conditions (including intra-layer and inter-layer connections), as shown in Figure 3. The results indicate that across all bands, the connection weights for AS-CE and AS-NC are almost identical, while the connection weights for the NAS Task are significantly lower than those for AS-CE and AS-NC. Statistical analyses revealed significant differences between NAS and AS tasks across all three bands. However, the weight difference between AS-CE and AS-NC was not significant (theta: *p* = 0.5054, alpha: *p* = 0.3254, beta: *p* = 0.4367).

### 3.2 Graph metrics of multi-layer networks

**Figure 4:**
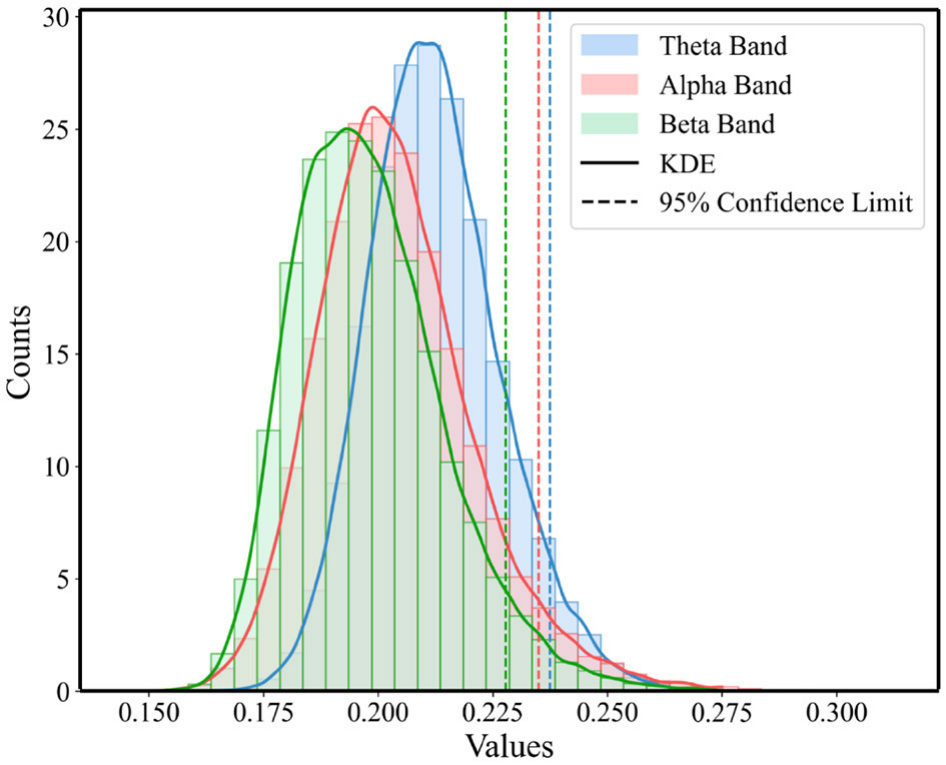
Statistical distribution of PLI value and the PDF for the three conditions across theta, alpha, and beta Bands.

To highlight those connections that are crucial for functional communication in the brain and to ensure the accuracy and effectiveness of the analysis, this study employed a method based on the statistical distribution of brain network connectivity matrices to determine the threshold for functional brain networks. For each band in Figure 2, we constructed a one-dimensional vector of PLI values for three super-adjacency matrices, which simultaneously consider the values of diagonal and off-diagonal blocks to equally consider connections within and between windows. Then, as illustrated in Figure 4, we analyzed the statistical distribution and Probability Density Function (PDF) of these PLI values. By calculating the 95% confidence boundary, we determined the threshold for the theta band to be 0.2375, for the alpha band to be 0.2349, and for the beta band to be 0.2278.

**Figure 5:**
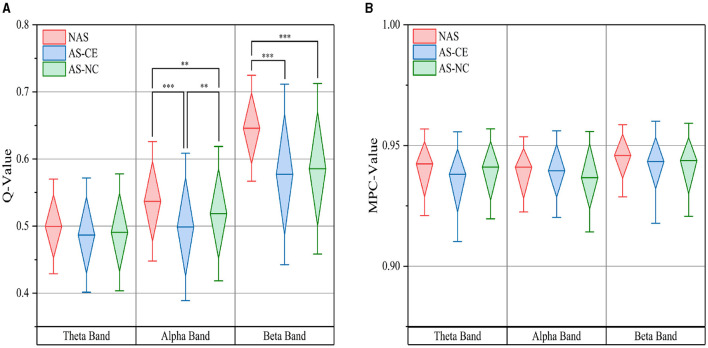
**(A)** illustrates the distribution of the Q-value, while **(B)** depicts the MPC value, both measured across theta, alpha, and beta bands under three task conditions. **p-value < 0.01, ***p-value < 0.001.

Based on thresholded weighted networks, we calculated three multi-layer network metrics: Q-value, MPC, and LLC. The Q-value results are displayed in Figure 5A using box plots, where the top and bottom boundaries represent the 25^th^ and 75^th^ percentiles, respectively, indicating the distribution of the central 50% of the data. Across all three bands, the Q-value for NAS tasks were found to be higher than those for AS tasks, with significant differences observed in the alpha and beta bands. Additionally, in the theta band, the distribution ranges of Q-value for the three task states were similar, showing no significant differences. A notable distinction was also identified in the alpha band between AS-CE and AS-NC tasks (*p* < 0.001). However, for MPC, as shown in Figure 5B, the values for the three tasks were similar and did not demonstrate any significant differences.

**Figure 6:**
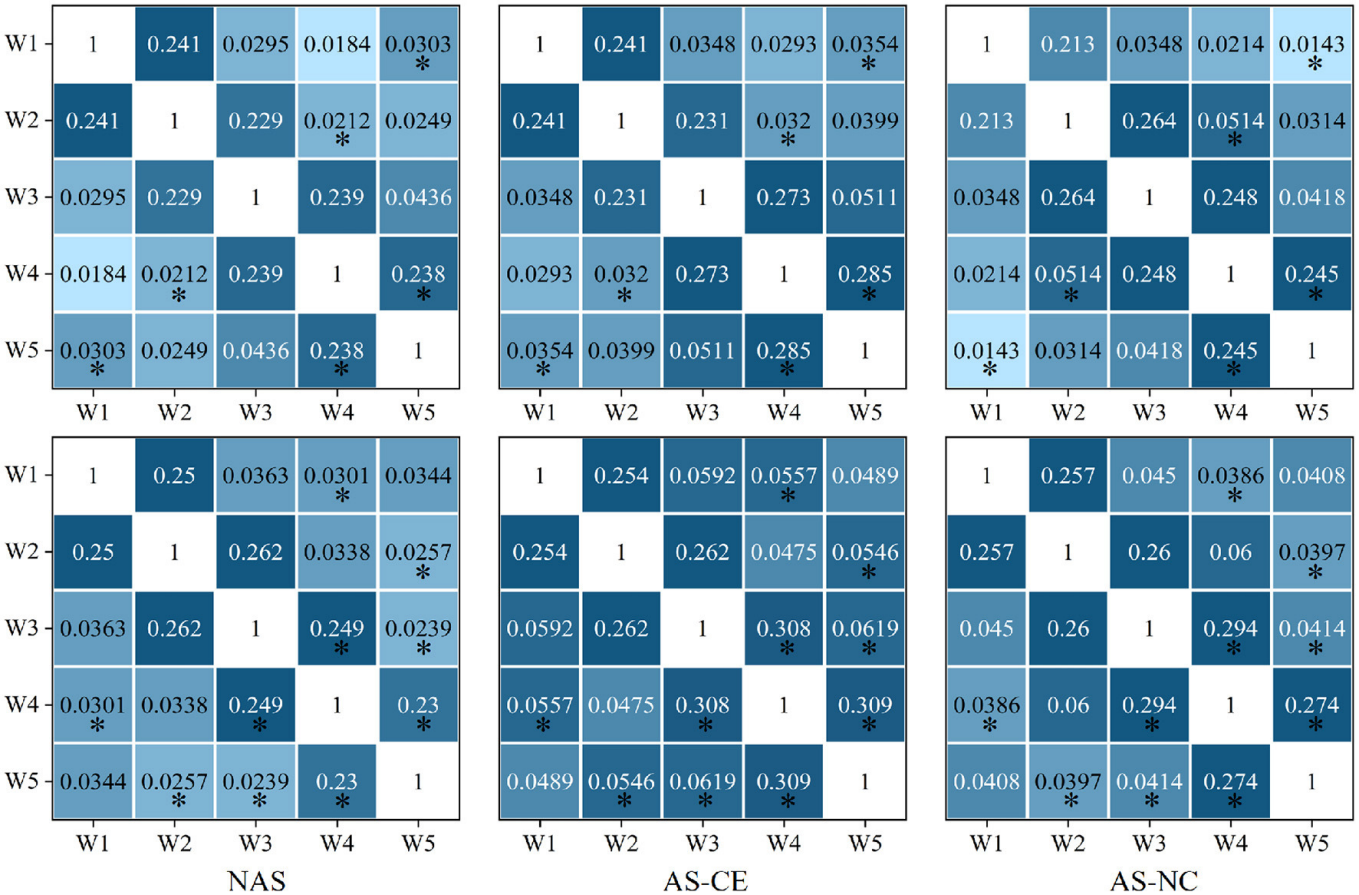
The LLC matrix displays numerical values representing LLC coefficients. “*” mark statistically significant differences between NAS and AS (including AS-CE and AS-NC), The top down is alpha and beta.

We calculated the distribution of LLC across different network layers under three task states in the theta, alpha, and beta bands. The observed LLC matrices exhibit symmetry and show higher LLC values between adjacent time windows, which gradually decrease with increasing time intervals. This indicates that the PLI connectivity strength of the brain network maintains a high level of similarity over short time periods, but gradually weakens over longer time scales. After conducting a detailed statistical analysis of the LLC matrices in the theta, alpha, and beta bands, no significant differences were found in the theta band. However, in the alpha band, significant differences in correlations were found between w1 and w5; w2 and w4; as well as between w4 and w5. In the beta band, significant differences were observed between W4 and each of W1 and W3; and between W5 and each of W2, W3, and W4. Figure 6 presents the distribution of LLC across different network layers under three task states in the alpha and beta bands.

### 3.3 Graph metrics of single-layer networks

#### 3.3.1 Analysis of local metrics

**Figure 7:**
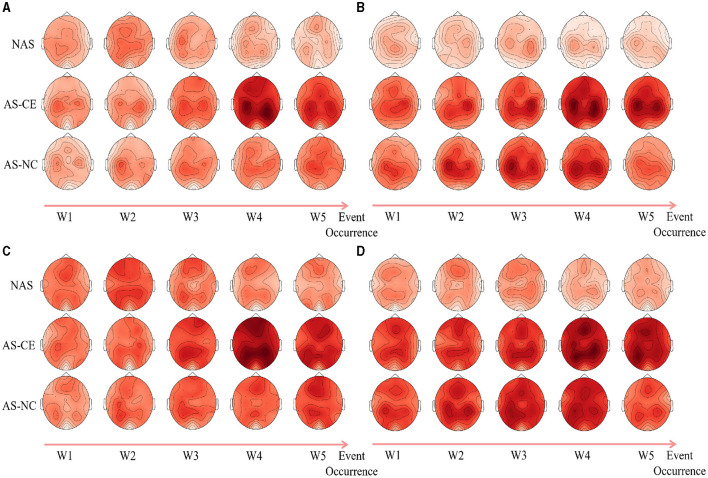
**(A, B)** Illustrate the topographical distribution of NS and E-loc in the Alpha band, while **(C, D)** depict the same for the Beta band. Each row corresponds to a distinct task state, while each column represents a specific time window. The intensity of the metric values is depicted through a gradient of color shades.

Following an analysis of local graph-theoretical metrics in single-layer networks, significant findings were observed in the NS and E-loc metrics within the Alpha and Beta bands, and did not show any significance in the remaining metrics. Figure 7 illustrates the distribution of these metrics across different task states (NAS, AS-CE, and AS-NC) and time windows (W1 to W5). Figures 7A, B depict the topographical distribution of NS and E-loc in the Alpha band, respectively. It is evident that the NS and E-loc distributions in NAS tasks are significantly lower than in AS tasks. Additionally, significant differences between AS-CE, and AS-NC tasks were observed in some channels during the time window W4 of the alpha band. Similarly, as shown in Figures 7C, D, NAS tasks in the Beta band also exhibited lower values. At time window W5, coinciding with the event occurrence, brain regions showed more intense activities compared to other time windows, with significant differences between AS-CE, and AS-NC tasks in some channels at W5 (Statistically significant channels listed in Table 1. For metrics that did not show statistical significance, please refer to the Supplementary Table S1).

**Table 1:** Statistically significant metrics utilized.

**Bands**	**Windows**	**Metrics**	**EEG channels**
Alpha	/	Q-value	/
		NS	Pz, P4, T6, FT7, CP4
	4	E-loc	Fp1, P3, P4, T6, CP4, CPz
		CC	/
Beta		NS	F3, Fz, C4, CP3, CP4
	5	E-loc	F3, P3, FC4
		CC	/

#### 3.3.2 Analysis of global metrics

**Figure 8:**
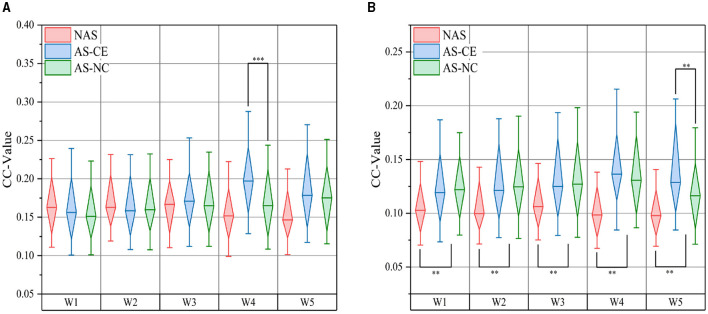
Comparison of CC: **(A)** presents comparisons in the alpha band, and **(B)** in the beta band. **p-value < 0.01, ***p-value < 0.001.

In the complex domain of multi-layer dynamic brain network research, global metrics play a pivotal role. We computed the CC and Ass coefficient for each time window within the theta, alpha, and beta bands. Figure 8 meticulously illustrates the variations in CC values across NAS, AS-CE, and AS-NC in the alpha and beta bands. Notably, in the beta band, significant statistical differences were observed in CC between NAS and AS for each time window. Of particular interest, significant differences between AS-CE and AS-NC were evident in the alpha band's W4 and the beta band's W5. Detailed data on global metrics can be found in Supplementary material.

### 3.4 Classification of alertness states

To further assess whether network metrics with significant differences could serve as potential biomarkers to effectively distinguish between AS-CE and AS-NC, thereby indicating the predictability of ollision perception in driving intuition under specific circumstances, we utilized three representative algorithms for classification: Support Vector Machine (SVM) with linear and radial basis function (RBF) kernels, based on statistical learning; Random Forest (RF), based on ensemble learning; and the K-Nearest Neighbors algorithm (KNN), based on Euclidean distance. These potential biomarkers were employed as inputs for these classifiers, with the biomarkers used listed in Table 1. To ensure unbiased and statistically significant results, the classifiers were evaluated using 5-fold cross-validation. Classification performance was quantified and assessed using the average accuracy, sensitivity, specificity, and precision, as detailed in Table 2, with linear kernel SVM demonstrating the best performance. In addition, we also conducted comparative analysis using features extracted only from a single-layer network without constructing a multi-layer network to evaluate the effectiveness of simpler network models in classification tasks.

**Table 2:** Classification outcomes corresponding to different classifiers.

	**JTF-MDBN features**				**Single-layer features**			
	**SVM-rbf**	**SVM-Linear**	**RF**	**KNN-3**	**SVM-rbf**	**SVM-Linear**	**RF**	**KNN-3**
Accuracy	81.5%	**87.5%**	87.2%	73.3%	69.8%	72.4%	74.1%	65.3%
Sensitivity	0.80	0.86	0.82	0.65	0.68	0.70	0.73	0.55
Specificity	0.83	0.89	0.92	0.82	0.70	0.77	0.76	0.70
Precision	0.82	0.88	0.91	0.78	0.69	0.75	0.73	0.68

The bold values signify the largest numerical results.

**Figure 9:**
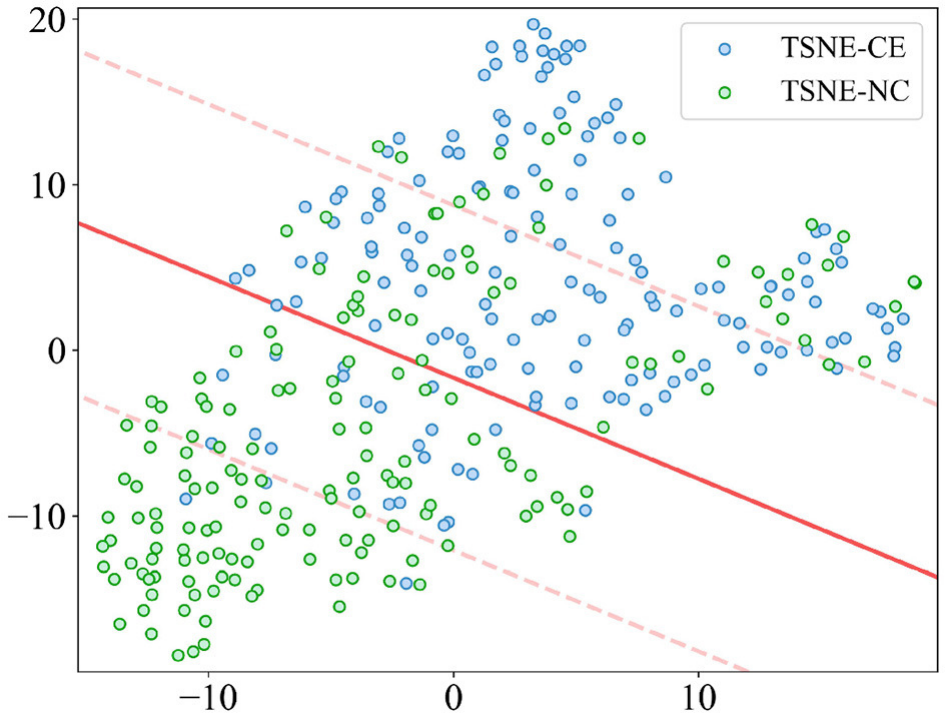
t-SNE visualization of AS differentiation between AS-CE and AS-NC.

The t-distributed Stochastic Neighbor Embedding (t-SNE) results, as depicted in Figure 9, are derived from a dimensionality reduction of 22 features aimed at distinguishing alertness states between AS-CE and AS-NC. The scatter plot demonstrates that, although there is some overlap between the two categories in the two-dimensional t-SNE space, a clear pattern along one of the axes suggests that these features have the potential for effective classification of the AS.

## 4 Discussion

In this study, we conducted an in-depth analysis of EEG-based multi-layer dynamic brain networks using PLI, with the aim of exploring driving intuition and anticipatory collision perception capabilities. Our findings reveal that intuition training enhances drivers' predictive intuitive responses to potential collision scenarios, concurrently facilitating the stabilization of brain network structures. By conducting a comprehensive analysis of various graph-theoretical metrics, we identified significant biomarkers in the context of intuitive driving, such as Q-value and NS in certain channels. We also adopted MPC and LLC to enhance our understanding of the brain's predictive ability in collision scenarios. These multi-layer network analysis methods have proven their effectiveness in EEG analysis of driving scenarios (Dang et al., 2020). These biomarkers effectively discriminate between AS-CE and AS-NC scenarios under the AS in the ITAP. This classification task holds practical importance for unveiling the underlying mechanisms of intuitive driving. Our results demonstrate significant differences in parameters of multi-layer and single-layer networks across different task states, particularly during AS tasks, offering new insights into understanding and predicting drivers' intuitive responses in potential collision scenarios.

### 4.1 Enhancement of intuitive driving skills through training

In this study, the role of intuition training is evaluated for its impact on enhancing individuals' intuitive response capabilities in complex driving tasks. We study the first five trials and the last five trials of the experimental process separately and constructed the JTF-MDBN as shown in Figure 2. We observe that brain network connectivity patterns in the ITAP are more stable and pronounced compared to the ITIP, especially in the intra-layer connections, it shows a stronger connection strength. This stability may stem from the enhanced functioning of task-related brain regions and neuroplastic changes induced by repetitive task execution (Chu et al., 2012). These findings provide evidence for the plasticity of intuitive abilities, supporting the notion that intuition is not only innate but can also be enhanced through appropriate training (Hogarth, 2001; Fellnhofer et al., 2023).

Given that the EEG data from the ITAP reflect a more mature and stable intuitive processing ability, our analysis is confined to this phase. The ITAP data more accurately represent the enhanced intuitive capabilities post-training, and the brain networks during the ITAP have adapted to the experimental environment, the data's stability and reliability are higher, thus making the analysis more representative and predictive.

### 4.2 Dynamics of brain network in driving intuition

We conducted a detailed analysis of the brain network structural features under NAS, AS-CE, and AS-NC. By establishing a hyperadjacency matrix, we observed that in all three bands, as shown in the ITAP brain network of Figure 2, the NAS task exhibited significantly lower PLI connectivity strength compared to the AS task. Specifically, in the AS task, the differences appeared after W3, with the AS-CE task showing stronger network activity in time window W4, persisting until W5, while the AS-NC task exhibited a gradual weakening trend in W5. This difference was particularly pronounced in the Beta band, potentially due to the correlation between the Beta frequency and human brain alertness (Braboszcz and Delorme, 2011). Additionally, as shown in Figure 7, the NS distribution indicated that the PLI connectivity strength in the NAS was lower than in the AS, and in the AS, the NS distribution on both sides of the brain roughly exhibited a symmetric distribution. In addition to the NS distribution mentioned above, in the single-layer metrics, both E-loc distribution and global metrics CC revealed features similar to those mentioned earlier.

Under the AS, the brain network exhibited significantly higher PLI connectivity strength compared to the NAS, which may be attributed to the increased information processing demands and rapid response requirements during alertness (Wang et al., 2021a). As shown in Figure 7, both NS and E-loc distribution maps, the prefrontal regions of the brain under the AS task gradually increased in the W3, W4, and W5 time windows before the collision occurred, and especially the left prefrontal region of the brain was significantly activated in the W4 time window of AS-CE. This result supports the idea that the left prefrontal cortex plays a key role in the regulation of alertness (Kim et al., 2017). According to several neuroscience studies, the left prefrontal cortex is recognized as one of the key brain regions regulating attention, decision making and executive functions. For example, in their study, Dosenbach et al. (2007) noted that the prefrontal cortex is involved in the regulation of higher cognitive processes such as planning, decision making, and task switching, which are essential for maintaining high alertness in complex environments. In addition, Corbetta and Shulman (2002)'s study emphasized the role of prefrontal vs. parietal networks in regulating attention and alertness, particularly in anticipating and responding to external stimuli. These studies echo our findings, suggesting that during the state of alertness, the PLI connectivity strength of brain networks increases, especially in prefrontal regions, possibly to enhance information processing and rapid response.

Surprisingly, when analyzing multi-layer graph-theoretical metrics, we observed a phenomenon different from the previously mentioned features. We observed that under the NAS, the value of Q-value was significantly higher than under the AS, as shown in Figure 5A. This difference may reflect the inherent structural differences in the brain network under different states. In the NAS, the brain may tend to maintain a higher degree of Q-value among various regions, indicating that different brain regions may engage in more independent processing, reducing interregional information exchange. Conversely, in the AS, due to the need for rapid response and stimulus processing, different brain regions may require closer cooperation, resulting in reduced Q-value. This tight network connectivity may facilitate rapid information transmission and integration, enabling the brain to effectively respond to urgent situations (Zhang et al., 2017). Therefore, the high Q-value under the NAS may reflect the independent processing characteristics of the brain in this state, while the low Q-value under the alertness state may be related to rapid response and decision-making processes.

### 4.3 Prediction of collision perception in driving intuition

We observed numerous significant features between the AS and NAS; however, given the presence of continuous risks in normal driving conditions and the necessity for drivers to maintain a high level of vigilance at all times, we chose not to classify these two states. Our research primarily focuses on investigating how changes in brain network features during the AS can predict a driver's perception of potential collision risks. It is essential to emphasize that the intuitive driver perception of danger referred to in this study is not a mystical sensation detached from scientific facts but is based on the driver's experience and a comprehensive assessment of various elements in the driving environment. This intuition can be acquired and enhanced through training and is not disconnected from scientific reality (Fellnhofer et al., 2023).

We performed classification using a combination of multi-layer network metrics, single-layer network local metrics, and global metrics. The results demonstrate that individuals can indeed anticipate potential dangers before they occur. In time windows W4 and W5 during AS, we identified several significant biomarkers (see Table 1) that contribute to distinguishing different AS outcomes. Our analysis indicates that in the W4 time window, participants may have formed clear expectations regarding the outcome of an impending collision event. This expectation may enhance the correlation between the W4 time window and its adjacent time windows, as shown in the analysis results of LLC Figure 6, with significant correlation between time windows near W4. Furthermore, key parameters within the W4 and W5 time windows, such as NS and E-loc, show statistically significant differences, highlighting the importance of these time windows in predicting dangers during the AS. We also conducted an in-depth analysis of Q-value, MPC and LLC metrics, which exhibit significant dynamic changes in the W4 and W5 time windows. By using these statistically significant biomarkers, we finally achieved an average classification accuracy of up to 87.5% in a linear kernel SVM classifier, showing the feasibility of using these biomarkers to predict collision hazards during driving, and the application of this technique to automated driver assistance systems will further enhance driving safety. In the comparative study, we further analyzed classification performance using only single-layer network features. As indicated in Table 2, the classification results from single-layer network features alone were suboptimal. This underscores the complexity of brain dynamics, where a multi-layered, integrative approach significantly outperforms single-layer static analysis. The brain's functional architecture appears too intricate to be effectively captured by static, single-layer analyses alone.

## 5 Conclusion

Our study successfully demonstrated the feasibility of intuition-based driving collision perception through an in-depth analysis of driving intuition and collision anticipation perception. This finding not only highlights the important role of intuition in dealing with emergency driving situations, but also confirms the validity and value of brain network analysis as a research tool. We found that during intuitive driving, the ITAP brain network showed a more stable and significant pattern of connectivity. Specifically, the multi-layer network during the ITAP showed significantly stronger intra-layer connections than inter-layer connections, especially in the W3, W4, and W5 time windows in the AS task. In terms of multi-layer network metrics, there were significant differences in Q-value across task states, especially in the Alpha and Beta bands. In addition, the LLC matrix revealed that brain network connection strengths remained similar in short time scales but gradually weakened with increasing time intervals, and among the single-layer network metrics, NS and E-loc showed significant differences in the Alpha and Beta bands. Among the single-layer global metrics, CC showed variations across task states, especially in Beta bands with significant differences between AS-CE and AS-NC. Finally, by using SVM with linear kernel, the average classification accuracy reaches up to 87.5%, demonstrating that biomarkers with statistical differences can be effective in detecting potential driving collisions.

What sets our research apart is that we proposed a novel multi-layer network analysis method JTF-MDBN that can simultaneously analyze driving intuition in different bands and continuous time windows. It is well known that different bands of EEG act functionally differently and that brain networks for cognitive processing in the brain change rapidly, making our proposed method well-suited for such a study. And we combine the features of multi-layer and single-layer brain networks to provide insights from both global and local. Multi-layer network analysis provides a richer understanding of brain network dynamics in the spatial domain than static methods. Single-layer brain network analysis reveals differences between states in more detail, providing critical information for identifying and categorizing driving states.

In our future work, we expect to design more realistic and diversified intuition-evoking experimental scenarios to fully evoke human intuitive responses. To further strengthen the credibility and depth of our research results, we will collect multimodal data. Additionally, we will utilize EEG equipment with a larger number of channels and more advanced EEG data analysis methods to study the intuitive ability in the human brain. We will continue to develop and refine network analysis methodologies based on neural network algorithms, aiming to enhance their applicability and effectiveness across datasets of varying sizes and complexities. Combined with the current analysis results, we will develop new biomarkers for intuition research, as well as study the associations between the brain regions that exhibit significance across different intuitive tasks, and further explore the connection between intrinsic intuitive mechanisms and external stimuli. Moreover, we will explore integrating human intuition with AI to enhance its capabilities to correctly handle unfamiliar and complex situations.

## Data availability statement

The datasets presented in this study can be found in online repositories. The names of the repository/repositories and accession number(s) can be found below: https://figshare.com/articles/dataset/Driving_with_Intuition_raw_data/3573888.

## Author contributions

JL: Data curation, Methodology, Validation, Writing – original draft, Writing – review & editing. ZW: Methodology, Supervision, Validation, Writing – review & editing. JH: Data curation, Validation, Visualization, Writing – review & editing. LZ: Supervision, Validation, Writing – review & editing.
