# Supplementary material for: EEG-based driving intuition and collision anticipation using joint temporal-frequency multi-layer dynamic brain network
Source: Front Neurosci. 2024 Jun 26;18:1421010. doi: 10.3389/fnins.2024.1421010 (PMC11233801; doi:10.3389/fnins.2024.1421010)
Supplement: Supplementary file 1 [file Data_Sheet_1.PDF]

Supplementary Material

1 SUPPLEMENTARY DATA

1.1 Distribution of Electrical Channels

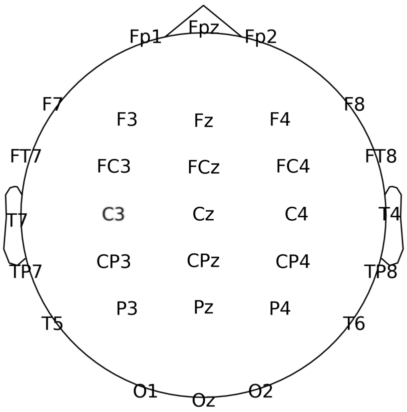

Figure S1. Distribution diagram of electrical channels

1.2 Single-layer Network Meatures

In this study, we employed various single-layer network metrics to analyze EEG data, reflecting the connectivity characteristics of brain networks at different levels. Below is a brief description of the metrics used:

Node Strength:$k_i^w = \sum_{j\in N} w_{ij}$

Path Length:$L^w = \frac{1}{n}\sum_{i\in N}\frac{\sum_{j\in N, j\neq i} d_{ij}^W}{n-1}$

Local Efficiency:$E_{loc}^w = \frac{1}{2}\sum_{i\in N}\frac{\sum_{j\leq eN,ji}(w_{ij}w_{ii}\left[d_{jh}^w(N_i)\right]^{-1})^{1/3}}{k_i(k_i-1)}$

Betweenness Centrality:$b_i = \frac{1}{(n-1)(n-2)}\sum_{h\neq j, j\neq Ni, j\neq i,}\frac{\rho_{ijj}(i)}{\rho_{ijj}}$

Eigenvector Centrality:$\lambda x^T = x^T A$

Clustering Coefficient:$C^w = \frac{1}{n}\sum_{i\in N}\frac{2t_i^W}{k_i(k_i-1)}$

Assortativity:$r^{\mathrm{w}} = \frac{l^{-1}\sum_{(i,j)\in L} w_{ij}k_i^{\mathrm{w}}k_j^{\mathrm{w}} - \left[l^{-1}\sum_{(i,j)\in L}\frac{1}{2}w_{ij}(k_i^{\mathrm{w}}+k_j^{\mathrm{w}})\right]^2}{l^{-1}\sum_{(i,j)\in L}\frac{1}{2}w_{ij}\left(\left(k_i^{\mathrm{w}}\right)^2+\left(k_j^{\mathrm{w}}\right)^2\right) - \left[l^{-1}\sum_{(i,j)\in L}\frac{1}{2}w_{ij}(k_i^{\mathrm{w}}+k_j^{\mathrm{w}})\right]^2}$

1.3 Results of single-layer network graph metrics

Table S1. Average of nodal metrics for single-layer networks.

Task Type	Metrics	W1	W2	W3	W4	W5
NAS	PL-θ	3.1222	3.1186	3.1365	3.1181	3.1045
	PL-α	3.1883	3.1769	3.1827	3.1863	3.1869
	PL-β	3.1663	3.1597	3.1654	3.1632	3.1627
	BC-θ	11.2756	11.0895	11.1884	10.9846	11.1473
	BC-α	7.9657	7.8409	7.9477	8.0354	7.8732
	BC-β	12.6204	12.8419	12.7482	12.7308	12.5955
	EC-θ	0.1628	0.1629	0.1629	0.1636	0.1630
	EC-α	0.1685	0.1687	0.1685	0.1685	0.1683
	EC-β	0.1622	0.1618	0.1621	0.1620	0.1622
AS-CE	PL-θ	3.1227	3.1203	3.1335	3.1026	3.0960
	PL-α	3.1750	3.1802	3.1795	3.1872	3.1853
	PL-β	3.1602	3.1583	3.1637	3.1641	3.1615
	BC-θ	11.3189	10.9515	10.9709	10.9678	11.7202
	BC-α	7.8695	7.9247	7.8876	7.8634	7.8202
	BC-β	12.1520	12.0139	12.1218	12. 1897	11.9828
	EC-θ	0.1632	0.1629	0. 1631	0.1634	0.1625
	EC-α	0.1685	0.1685	0.1686	0.1685	0.1687
	EC-β	0.1622	0.1625	0.1624	0.1624	0.1625
AS-NC	PL-θ	3.1202	3.1238	3.1292	3.1091	3.0962
	PL-α	3.1810	3.1825	3.1808	3.1893	3.1815
	PL-β	3.1657	3.1611	3.1620	3.1598	3.1613
	BC-θ	11.0173	10.9952	10.6098	10.7520	11.4299
	BC-α	7.8159	7.9705	7.9039	7.8538	7.9906
	BC-β	12.1895	12.1716	12.1160	12.1232	12.5532
	EC-θ	0.1633	0.1627	0.1630	0.1632	0.1627
	EC-α	0.1687	0.1684	0.1687	0.1680	0.1684
	EC-β	0.1624	0.1623	0.1627	0.1625	0.1620

Table S2. Average of global metrics for single-layer networks.

Task Type	Metrics	W1	W2	W3	W4	W5
NAS	CC-θ	0.1768	0.1762	0.1171	0.1758	0.1775
	CC-α	0.1686	0.1753	0.1677	0.1607	0.1571
	CC-β	0.1093	0.1072	0.1108	0.1027	0.1050
	Ass-θ	-0.1148	-0.1103	-0.1148	-0.1090	-0.0947
	Ass-α	-0.1094	-0.0902	-0.1011	-0.1043	-0.0867
	Ass-β	-0.0947	-0.0942	-0.0975	-0.0878	-0.1012
AS-CE	CC-θ	0.1754	0.1812	0.1791	0.1773	0.1690
	CC-α	0.1701	0.1697	0.1827	0.2081	0.1938
	CC-β	0.1302	0.1326	0.1364	0.1500	0.1454
	Ass-θ	-0.1115	-0.0971	-0.1118	-0.1082	-0.1061
	Ass-α	-0.0994	-0.0931	-0.0990	-0.0947	-0.0929
	Ass-β	-0.0965	-0.1006	-0.0851	-0.0853	-0.0913
AS-NC	CC-θ	0.1781	0.1803	0.1878	0.1881	0.1718
	CC-α	0.1622	0.1700	0.1734	0.1760	0.1834
	CC-β	0.1273	0.1334	0.1380	0.1402	0.1254
	Ass-θ	-0.1086	-0.0969	-0.1027	-0.1047	-0.0993
	Ass-α	-0.0978	-0.1070	-0.1046	-0.0984	-0.1043
	Ass-β	-0.1071	-0.1018	-0.0877	-0.0973	-0.1007
